# Using morphological attributes for the fast assessment of nutritional responses of Buddhist pine (*Podocarpus macrophyllus* [Thunb.] D. Don) seedlings to exponential fertilization

**DOI:** 10.1371/journal.pone.0225708

**Published:** 2019-12-09

**Authors:** Liang Xu, Xie Zhang, Duhai Zhang, Hongxu Wei, Jia Guo

**Affiliations:** 1 Zhejiang Academy of Forestry, Hangzhou, Zhejiang, China; 2 Hunan Academy of Forestry, Changsha, Hunan, China; 3 Northeast Institute of Geography and Agroecology, Chinese Academy of Sciences, Changchun, Jilin, China; 4 Chengbang Ecological Environment Limited Liability Company, Hangzhou, Zhejiang, China; Centro de Edafologia y Biologia Aplicada del Segura, SPAIN

## Abstract

Culturing slowly growing tree seedlings is a potential approach for managing the conflict between the increasing demand for ornamental stock and the decreasing area of farmlands due to urbanization. In this study, Buddhist pine (*Podocarpus macrophyllus* [Thunb.] D. Don) seedlings were raised in multishelves with light-emitting diode lighting in the spectrum of 17:75:8 (red:green:blue) at 190–320 μmol m^-2^ s^-1^ with controlled temperature and relative humidity at 19.5°C and 60%, respectively. Seedlings were fed by exponential fertilization (EF) (nitrogen [N]-phosphorus [P]_2_O_5_-K_2_O, 10-7-9) at eight rates of 0 (control), 20 (E20), 40 (E40), 60 (E60), 80 (E80), 100 (E100), 120 (E120), and 140 (E140) mg N seedling^-1^ for four months through 16 fertilizer applications. The nutritional responses of Buddhist pine seedlings can be identified and classified into various stages in response to increasing doses, up to and over 120 N seedling^-1^. Morphological traits, i.e., the green color index and leaf area (LA) obtained by digital analysis and the fine root growth, all remained constant in response to doses that induced steady nutrient loading. LA had a positive relationship with most of the nutritional parameters. A dose range between 60 and 120 mg N seedling^-1^ was recommended for the culture of Buddhist pine seedlings. At this range of fertilizer doses, measuring the leaf area through digital scanning can easily and rapidly indicate the inherent nutrient status of the seedlings.

## Introduction

Urbanization is one of the most critical forces driving land use change on Earth. Urban forest is a basic greening infrastructure that is needed to improve regional environmental quality and provide a restorative setting for humans [1,2]. Slow-growing ornamental trees are less used than faster-growing varieties due to the high cost of long-term production and their slow establishment. However, they are as important as fast-growing species because including these trees promotes biodiversity and improves the scenery of the urban landscape.

Newly planted trees in an urbanized area undergo multiple challenges before establishment. During initial tree development, soil drought and nutrient deficiencies are the factors that most likely cause mortality [3]. Thus, transplanting ornamental trees can be controlled by watering and fertilizing to help the trees cope with transplant shock. The cost of maintaining urban forests can be reduced if transplant shock can be overcome by the inherent nutrient utilization of the seedlings [4]. However, the fertilization of slowly growing ornamental seedlings before transplantation is limited by their low efficiency of nutrient uptake and utilization. This suggests the importance of developing a culture regime to rapidly promote the quality of slowly growing ornamental tree seedlings.

Seedling quality is the core of any successful forest restoration program because it is related to tree survival and performance after transplantation [5]. A quality seedling shows multiple attributes within some optimum ranges that together determine the transplant success [6]. The formation of inherent nutrient reserves at transplant is one of the key physiological attributes of a high-quality seedling, which can be enhanced by nutrient loading during nursery culture [7]. It is widely believed that the presence of an abundant nutrient reserve in seedlings at transplant could contribute to overcoming early-stage competition with weeds, and relevant evidence to support this argument is still accumulating [8–11]. Previous studies, however, have mostly been conducted in traditional nurseries where environmental factors can only be roughly controlled. Recent studies found that under greenhouse conditions where the lighting intensity, light spectrum, moisture, and temperature were adjusted, slowly growing ornamental tree seedlings can be efficiently grown to the desired morphology for sufficient nutrient storage through nutrient loading in the short term [12–15]. Therefore, it is necessary to further test the effect of nutrient loading on slowly growing ornamental tree seedlings under controlled conditions where environmental factors are better managed.

Continuous land transformation through urbanization is reducing the rural space for cultivated land. This has also stimulated a change from the conventional regime of plant production to the newly developed approach that target seedlings are produced in a shortened term using a small space. The regime of seedling culture in multishelves where physical factors are well controlled to a specific growing condition could solve the problem of cultivated land limitation in raising plants [16]. Innovation in hardware manipulation has never stopped moving forward, but the focus on the plant itself has been neglected [17]. Under controlled conditions, either the quality or the stature of vegetable crops can be easily modified by controlling the environmental factors because their destination is to be harvested. However, for forest crops, enhancing their inherent quality so that they can adapt to field conditions is more important than shaping a desired size. This is because plant factory can produce trees that survive and establish in the field over the whole process from nursery culture to transplant.

The current system of tree seedling quality evaluation is established on the basis of forecasting transplanted performance by measuring plant attributes at the end of the nursery stage [5,6]. This approach originated in the mid-twentieth century and has been used for several decades. However, the current forecast models for seedling quality need to be frequently amended to account for the attributes of a wide range of tree species in complicated field conditions in different biomes (see [5]). In addition, the success of forecasting seedling quality using plant traits largely depends on transplant performance in the field, which can be determined by the inherent nutrient reserve of the seedling. Under controlled conditions, seedling morphology can be shaped as desired by regulating environmental factors at any time. Therefore, identifying the relationship between plant traits and inherent nutrient content is critical for quickly and precisely evaluating seedling quality.

The development of digital image analysis techniques has enabled the precise evaluation of nutrient concentration by scanning leaf traits in crop plants [18,19]. This technique has not yet been used on tree species for the purpose of forecasting inherent nutrient status. In this study, Buddhist pine (*Podocarpus macrophyllus* [Thunb.] D. Don) seedlings were cultured as slowly growing ornamental tree material in a greenhouse where light, temperature, moisture, and substrate were all controlled to optimum levels. A range of doses of fertilizers were applied to create a gradient in nutrient status. Both traditional growth measurements of seedling height and collar-diameter and those from the digital analysis of leaf images were measured to detect their relationships with the inherent nutritional parameters. It was hypothesized that (i) seedlings with different nutritional statuses will have different digital analyses on scanned leaves that directly correlate with nutritional status of target seedlings, and (ii) green degree of an image and topographic estimate of projected area for leaves will be the two parameters that have tight correlation with nutrient concentration.

## Materials and methods

### Seedling material

Buddhist pine seeds were obtained from trees at Hangzhou (30^o^10’ N, 120^o^20’ E), Zhejiang, China. Seeds were sterilized by potassium (K) permanganate (0.5%, w/w), soaked for 12 h, and sown at 0.5 cm deep in sands with a moisture content of 80% at a constant temperature of 22°C in the Laboratory of Combined Manipulation of Illumination and Fertility on Plant Growth (43°48.6’ N, 125°22.8’ E) (Zhilunpudao Agric. S&T Ltd., Changchun, China). During germination, seeds were kept at a relative humidity (RH) of 80% and a temperature of 28°C. Germinated seeds were moved to planting tray cells (approximately 0.2 L; 7 cm × 13 cm, top-diameter × height) filled with a commercial substrate of peat, spent-mushroom residue, and perlite (55:20:25, v/v/v) (Mashiro-Dust^TM^, Zhiluntuowei Agric. For. S&T Ltd., Changchun, Jilin, China). Thirty-two planting cells were arranged in a seedling tray with 4 × 8 spacing. Initial substrate properties were determined prior to this experiment. Ammonium and nitrate nitrogen (N) concentrations were determined by continuous flow analysis [20]. Available phosphorus (P) was determined by the Olsen method [21]. Organic matter, pH, and EC were determined according to the method of Wei *et al*. [22]. The initial substrate had an ammonium N content of 79.99±8.23 mg kg^-1^ (three replicated observations; the same below), nitrate N content of 1.67±0.78 g kg^-1^, available P content of 0.36±0.05 g kg^-1^, organic matter content of 12.9±0.15%, pH of 4.82±0.02, and electrical conductivity (EC) of 1.62±0.15 mS cm^-1^.

### Experimental conditions

Sunlight was blocked thoroughly by blackout curtains. The illumination for seedling growth was supplied solely by light-emitting diodes (LEDs) of approximately white color. The photosynthetic photon flux rate (PPFD) ranged between 190 (at the beginning) and 320 μmol m^-2^ s^-1^ (at the end) in the red (R; 600–700 nm): green (G; 500–600 nm): blue (B; 400–500 nm) spectrum at 17:75:8 in the range of visible light wavelengths between 350 and 800 nm. This lighting condition has been proven to be sufficient to support the growth of Buddhist pine seedlings [23]. LED diodes were embedded in a panel with a size of 0.4 m × 1.2 m. The electric currency of four LED panels was controlled by a common transformer to regulate light intensity and spectra to the desired levels. The temperature was controlled between 13.6°C and 28°C with an average of 19.5±5.3°C by an air conditioner. The relative humidity (RH) was maintained by a humidifier when necessary in a range of 60% to 90%.

### Seedling culture

Trays were placed in watering tanks located on the bases of metallic frames. Five metallic frames (height × width × length, 2 m × 0.4 m × 1.2 m) were employed as five replicate blocks containing varied treatments. Each frame consisted of four culture cells (height × width × length, 0.4 m × 0.4 m × 1.2 m). A LED panel (length × width, 1.2 m × 0.4 m) was fixed to the ceiling of each frame cell. Therefore, the initial distance between the LED panel and the seedlings was 27 cm, with the frame height of 40 cm and the tray height of 13 cm. The height of 27 cm also enabled the initial seedling shoot elongation. Based on previous studies [10,13], the daily photoperiod was set to 18 h from 6:00 a.m. to 24:00 p.m. This photoperiod was used for the culture of coniferous seedlings from different latitudinal populations [13,14,24].

### Fertilizer treatments

The experiment was conducted as a random block design with eight EF treatments in five replicated blocks (*n* = 5). Each block was assigned a growing frame. Eight seedling trays were randomly placed on a shelf of a culture cell to receive one of eight exponential fertilization (EF) rates of 0 (control), 20 (E20), 40 (E40), 60 (E60), 80 (E80), 100 (E100), 120 (E120), and 140 (E140) mg N seedling^-1^. This fertilization rate range covered, but was not limited to, that reported in a previous study on this species [25]. All fertilizers (N-P_2_O_5_-K_2_O, 10-7-9) were delivered through 16 applications once a week over four months, during which the control seedlings only received distilled water at each application. EF was commenced according to the N addition formulation used by Duan *et al*. [9] and Wei *et al*. [25]:

$$N_{T}=N_{S}(e^{rt}-1)$$ (1)

where *r* is the relative addition rate required to increase *N*_*S*_ to a final N content (*N*_*T*_ + *N*_*S*_) over the number of fertilizer applications (*t* = 16). *N*_*S*_ and *N*_*T*_ are the initial N content in seedlings and the desired amount to be added, respectively. In our study, *N*_*S*_ was set as 1.4 mg N seedling^-1^ according to Wei *et al*. [25]. The amount of fertilizer applied to seedlings at each application (*N*_*t*_) was computed as [9]:

$$N_{t}=N_{S}(e^{rt}-1)-N_{t-1}$$ (2)

where *N*_t-1_ is the cumulative amount of N added to seedlings, including the most recent application. One fertilized control treatment was also added.

### Seedling sampling, measurement and chemical analysis

Seedlings were sampled one week after the final application of fertilizer. Eight seedlings were randomly sampled from a tray as one bulk sample. Five bulk samples of seedlings were taken from five replicated blocks. Sampled seedlings had their roots cleaned of substrate and were then measured for height and root-collar diameter (RCD). Thereafter, four seedlings were randomly chosen and bulked as a group to measure their biomass, N concentration, and P concentration. The sampled seedlings were divided into shoot and root parts, and both parts were oven-dried at 70°C for 72 h, measured for dry weight (biomass), ground to powder, and used for digestion. The root to shoot biomass ratio (R/S) was calculated using the oven-dried weight data. A sample of 0.2 g dried power was digested in 5 ml of H_2_SO_4_-H_2_O_2_ (7/3, v/v). Fully digested samples were collected in plastic bottles at a volume of 50 ml and used for chemical analysis two days later. N and P were analyzed by the method of Wei *et al*. [12]. The N concentration was determined using the Kjeldahl method (UDK 152 automatic N analyzer, VELP^®^ Co., Usmate, MB, Italy), and the P concentration was determined by ICP-OES (Vista-MPX, Varian^®^, USA).

The other four seedlings were sampled for their needled leaves and fine roots. Leaves were cleaned of fertilizer with distilled water and carefully excised from the stem. Fine roots were defined as white roots growing around the tip of the taproot with a diameter of less than 1 mm. We focused on fine roots because they relate to root quality assessment and contribute more to lateral root growth than taproot [26]. Fine roots were rinsed and carefully cut off. Eight pieces of leaves were randomly chosen from the middle position of the stem and excised. Four groups of fine roots were excised from the four seedlings. Both leaves and roots were placed on the panel of a scanner (HP Deskjet 1510 scanner, HP Inc., Palo Alto, CA, USA) and scanned to generate a projected image at a resolution of 118.11 pixels cm^-1^. Scanned root images were analyzed by WinRHIZO software (Regent Ltd., Alberta, Canada) to measure fine root length, surface area, and tip number. Scanned leaf images were further processed using Photoshop software (ver. 8.0, Adobe^®^ Systems Incorporated Inc., San Jose, California, USA) by deleting all background colors, then reading for the histogram information. The average score for all pixels through the green-color channel was recorded as the green color index (GCI) of the leaves. Furthermore, leaf area (LA) can be calculated by the following model:

$$LA=\frac{{Pixel}_{all}}{{Res.}^{2}\times {Leaf}_{number}}$$ (3)

where *Pixel*_*all*_ is the whole pixel value for the projected area of 32 leaves; *Res*. is the resolution of the scanned image, which was 118.11 pixels cm^-1^; and *Leaf*_*number*_ is the number of leaves (*n* = 32).

### Statistical analysis

Data were analyzed using SAS (ver. 9.4 64-bit, SAS Institute Inc., NC, USA). A normality test was conducted on the data for each parameter, and no transformation was necessary. The effect of EF on seedling parameters was tested by analysis of variance (ANOVA). When effects were significant, the means were ranked according to Tukey’s studentized range test at the *α* = 0.05 level. Vector analysis was employed to diagnose the nutritional state of N and P in the shoot part according to the methodology used by Li *et al*. [14]. Pearson correlation was employed to analyze the relationship between the two leaf indices (GCI and LA) and the other variables.

## Results

### Seedling growth and morphology

**Fig 1:**
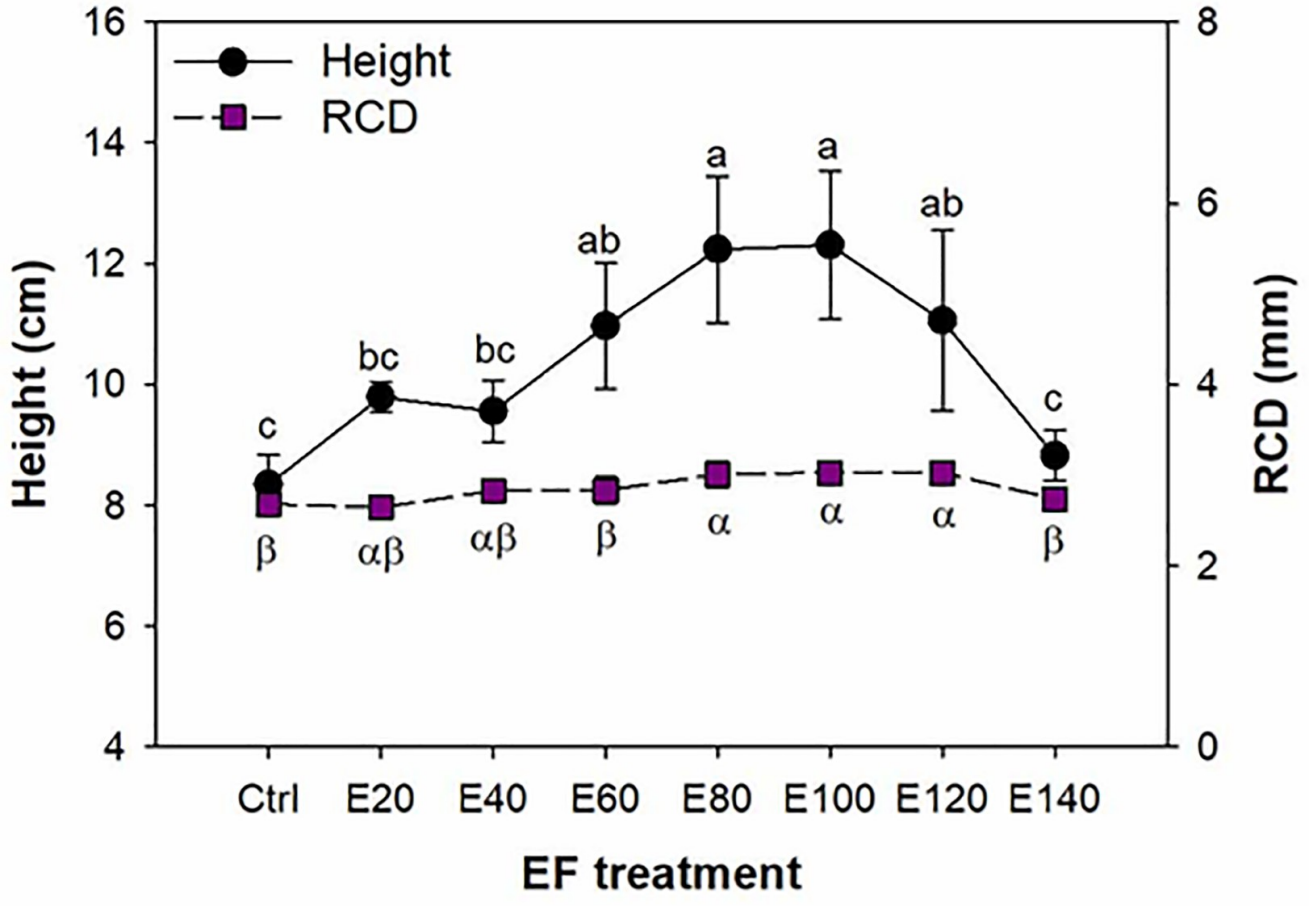
Height and root-collar diameter (RCD) in Buddhist pine (*Podocarpus macrophyllus* [Thunb.] D. Don) seedlings exposed to exponential fertilization (EF) treatments. EF was applied at eight rates: 0 (control), 20 (E20), 40 (E40), 60 (E60), 80 (E80), 100 (E100), 120 (E120), and 140 (E140) mg N seedling^-1^. Values are mean ± Standard error. Different lower-case letters indicate significant differences between fertilization treatments for plant height; Latin-letters indicate significant differences between fertilization treatments for RCD.

Seedling height did not significantly change with the increase in nutrient supply until the E60 treatment (Fig 1). Seedling height was not different among the E60, E80, E100, and E120 treatments (Fig 1). Height in the E140 treatment declined compared to that in the E120 treatment (*F*_7,32_ = 10.81; *P*<0.0001), but it was not significantly different from that in the control seedlings. With the increase in the EF rate, RCD was unchanged from the control to the E60 treatment, was higher in the E80, E100, and E120 treatments, and declined in the E140 treatment (*F*_7,32_ = 9.75; *P*<0.0001).

With the increase in the EF rate, root length, surface area, and tip number all increased in the E60 treatment through the E120 treatment and declined in the E140 treatment (Table 1). Among EF treatments, the E120 treatment resulted in the longest fine roots (*F*_7,32_ = 9.90; *P*<0.0001), the highest root surface area (*F*_7,32_ = 7.05; *P*<0.0001), and the highest root-tip number (*F*_7,32_ = 10.46; *P*<0.0001).

**Table 1:** Root morphology in *Podocarpus macrophyllus* seedlings cultured by exponential fertilization at rates of 0 (Control), 20 (E20), 40 (E40), 60 (E60), 80 (E80), 100 (E100), 120 (E120), and 140 (E140) mg N unit^-1^.

Treatment	*n*	Root length (cm)	Root surface area (cm^2^)	Root tip number (×100)
Control	5	351.97±26.82c	140.70±11.46d	1.06±0.06c
E20	5	479.55±129.86bc	186.99±53.10bcd	1.49±0.39bc
E40	5	444.24±70.02c	180.24±44.19cd	1.40±0.16bc
E60	5	690.76±187.29ab	287.24±94.64abc	1.77±0.41ab
E80	5	498.65±65.65bc	214.24±37.82abcd	1.42±0.17bc
E100	5	695.15±111.45ab	319.00±85.19ab	1.96±0.17ab
E120	5	778.78±131.60a	333.00±76.18a	2.13±0.31a
E140	5	347.32±45.75c	141.76±24.83d	1.03±0.12c

Different letters in a column indicate significant differences by Tukey’s test at the 0.05 level.

### Biomass accumulation and N and P uptake

**Fig 2:**
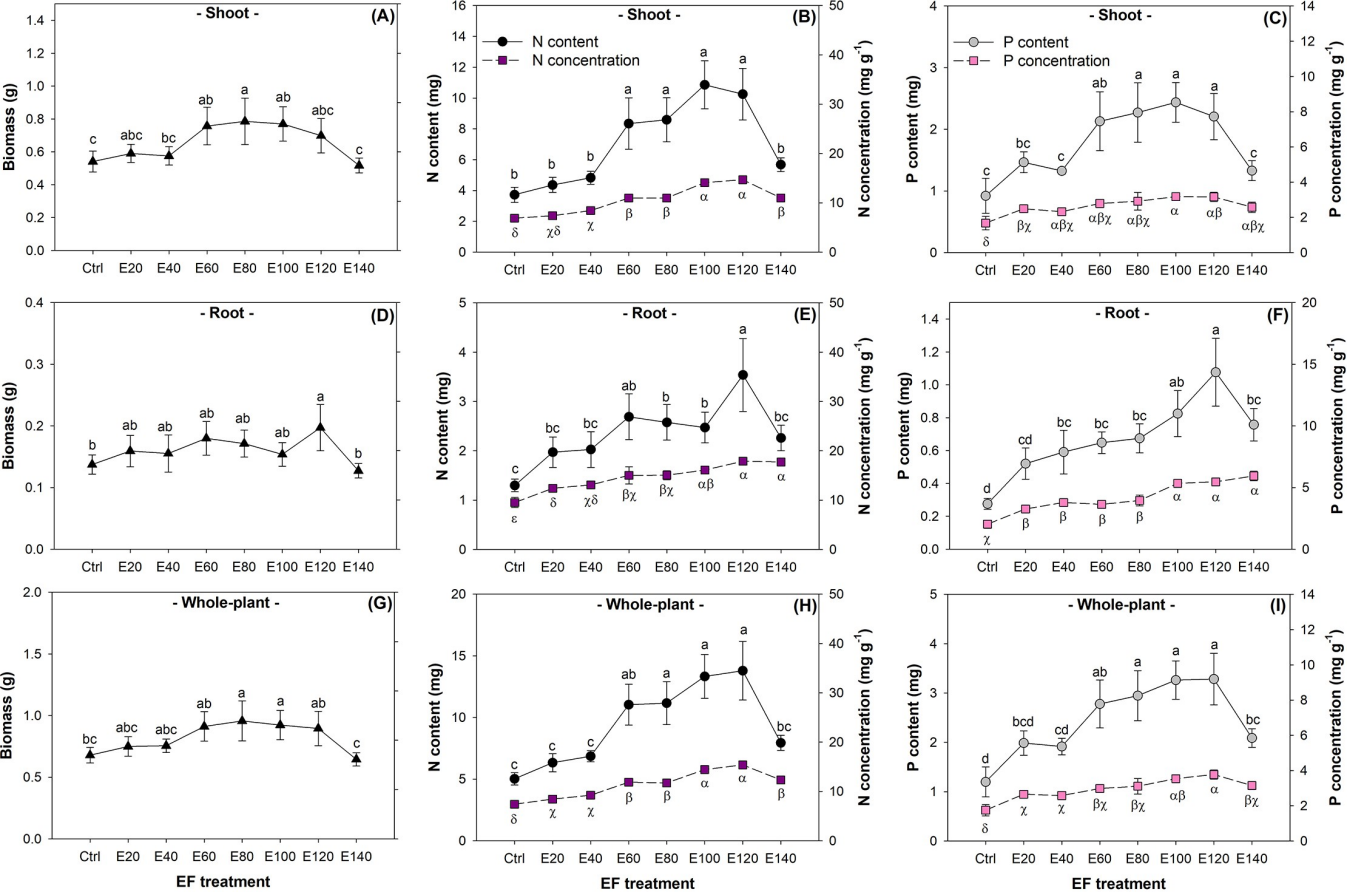
Biomass, nitrogen (N) content and concentration, and phosphorus (P) content and concentration in Buddhist pine (*Podocarpus macrophyllus* [Thunb.] D. Don) seedlings exposed to exponential fertilization (EF) treatments. EF was applied at eight rates: 0 (control), 20 (E20), 40 (E40), 60 (E60), 80 (E80), 100 (E100), 120 (E120), and 140 (E140) mg N seedling^-1^. Different letters present significant differences according to Tukey’s test at the 0.05 level. Values are mean ± Standard error. Different lower-case letters indicate significant differences between fertilization treatments for plant biomass and nutrient content; Latin-letters indicate significant differences between fertilization treatments for nutrient concentration.

Shoot biomass increased by approximately 25–29% with the increase in the EF rate from the E60 treatment to the E100 treatment compared to that in the control (*F*_7,32_ = 6.18; *P* = 0.0001) (Fig 2A). The shoot N content increased from the E60 treatment to the E120 treatment compared to those in the control and the E20 and E40 treatments (*F*_7,32_ = 24.04; *P*<0.0001), while the N concentration increased starting with the E40 treatment compared to that in the control (*F*_7,32_ = 129.85; *P*<0.0001) (Fig 2B). The shoot P content responded to the EF treatment with a similar trend as that of the shoot N content (*F*_7,32_ = 12.85; *P*<0.0001), but the shoot P concentration started to increase with the E20 treatment (*F*_7,32_ = 11.17; *P*<0.0001) (Fig 2C).

Root biomass remained unchanged from the E20 to the E100 treatments and increased in the E120 treatment compared to that in the control (*F*_7,32_ = 3.50; *P* = 0.0067) (Fig 2D). The root N content was higher from the E60 treatment to the E120 treatment compared to that in the control (*F*_7,32_ = 11.08; *P*<0.0001), while the root N concentration rose from the control to the E140 treatment (*F*_7,32_ = 42.22; *P*<0.0001) (Fig 2E). The root P content increased from the control to the E120 treatment, which resulted in the highest response to the increase in the EF rate (*F*_7,32_ = 16.59; *P*<0.0001), while the root P concentration also increased from the control to the E100 treatment (*F*_7,32_ = 66.32; *P*<0.0001) (Fig 2F).

At the whole-plant scale, biomass did not change significantly in the E20, E40, or E60 treatment but was higher in the E80 and E100 treatments compared to that in the control (*F*_7,32_ = 5.79; *P* = 0.0002) (Fig 2G). Both the N and P contents were higher in the E60 to E120 treatments, and the N and P concentrations were highest in the E100 and E120 treatments (Fig 2H and 2I).

### Vector analysis

**Fig 3:**
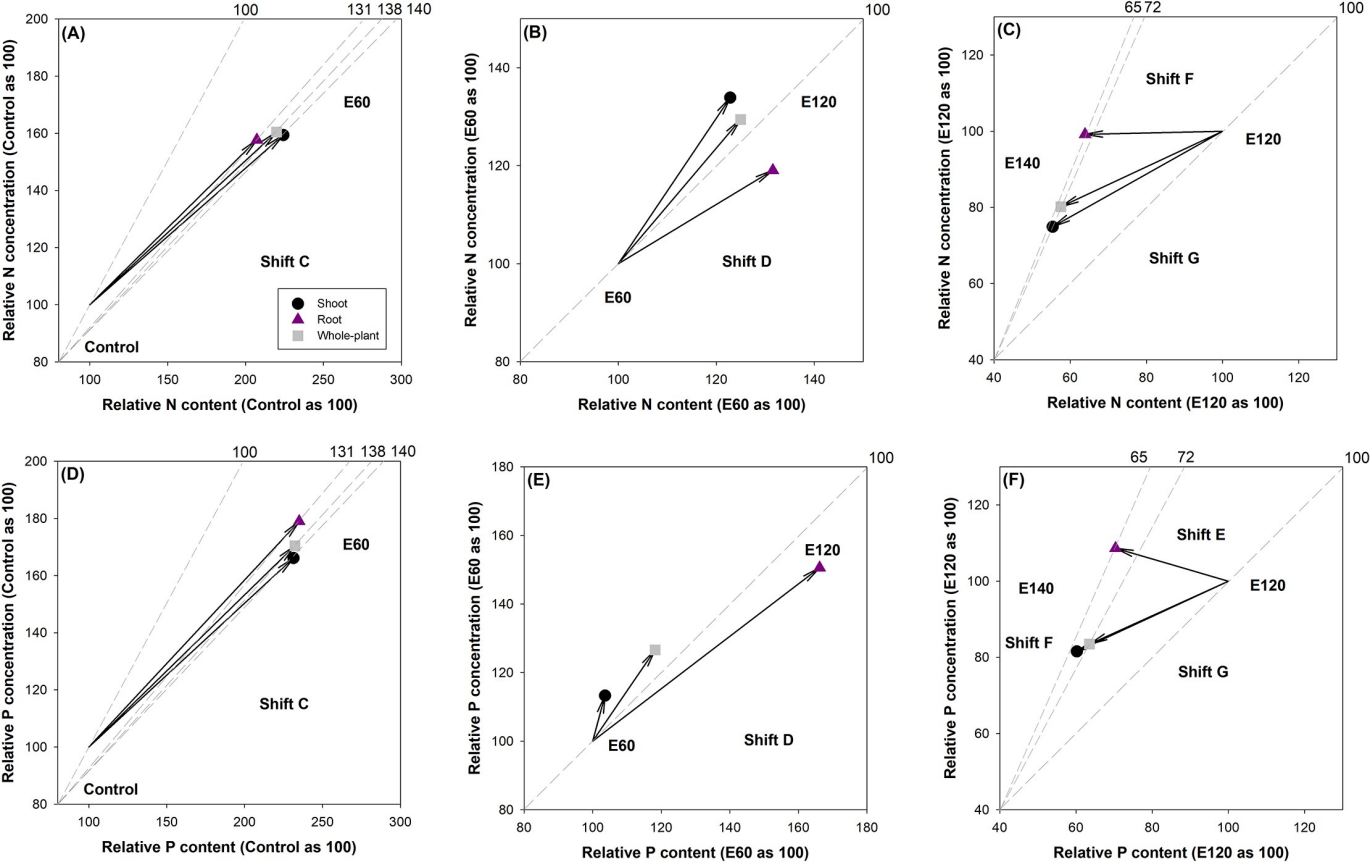
Vector analysis of the relative N and P statuses in Buddhist pine (*Podocarpus macrophyllus* [Thunb.] D. Don) seedlings exposed to exponential fertilization (EF) treatments. EF was applied at eight rates: 0 (control), 60 (E60), 120 (E120), and 140 (E140) mg N seedling^-1^. Vector shifts indicate the relative nutrient status as given by Salifu and Timmer (2003). Shift C, nutrient deficiency; Shift D, luxury nutrient consumption; Shift E & F, excessive nutrient toxicity; Shift G, excessive nutrient depletion.

According to the synthesized results for biomass, N and P content, and N and P concentrations, these factors appeared to be grouped into three stages separated by the E60, E120, and E140 treatments. Therefore, the relative nutritional status of the seedlings can be analyzed among these treatments (Fig 3A–3F). Relative to the control, biomass, N and P content, and N and P concentrations increased in the E60 treatment at all three scales (shoot, root, and whole-plant); hence, both N and P statuses in the E60 treatment can be characterized as countering the nutrient deficiency in the control (Fig 3A and 3D). Relative to the E60 treatment, both N and P contents and concentrations increased in the E120 treatment, but biomass was not significantly different between these two treatments. These symptoms can be classified as the luxury consumption state between the two other treatments (Fig 3B and 3E). Relative to the E120 treatment, the E140 treatment resulted in lower N and P content and concentrations in the shoot and the whole plant without significantly changing biomass; this state was characterized as relative nutrient depletion (Fig 3C and 3F). In roots, both biomass and N and P content declined in the E140 treatment relative to those in the E120 treatment; thus, the E140 treatment can be characterized as the state of relative nutrient excess (Fig 3C and 3F).

### Leaf trait and biomass allocation

With the increase in the EF rate, GCI showed the tendency of an inverse parabola, with the lowest value in the E100 treatment (Table 2). In contrast, LA increased from the control to the E100 treatment and declined through the E120 treatment to the E140 treatment. The root to shoot biomass ratio (R/S) tended to be highest in the E120 treatment, which was higher than that in the E100 treatment by 40%.

**Table 2:** Leaf green color index (GCI), leaf area (LA), and root to shoot ratio (R/S) in *Podocarpus macrophyllus* seedlings cultured by exponential fertilization at rates of 0 (Control), 20 (E20), 40 (E40), 60 (E60), 80 (E80), 100 (E100), 120 (E120), and 140 (E140) mg N unit^-1^.

Treatment	*n*	GCI	LA	R/S
Control	5	251.53±0.57a	16.87±1.49c	0.26±0.04ab
E20	5	250.96±0.18abc	19.12±0.47abc	0.27±0.02ab
E40	5	250.64±0.31bc	17.99±1.20abc	0.27±0.05ab
E60	5	250.93±0.14abc	19.09±1.05abc	0.24±0.02ab
E80	5	251.06±0.23abc	19.73±1.15ab	0.22±0.02ab
E100	5	250.33±0.26c	20.66±1.30a	0.20±0.02b
E120	5	250.64±0.47bc	19.50±1.95abc	0.28±0.03a
E140	5	251.31±0.32ab	17.30±1.08bc	0.25±0.02ab

Different letters in a column indicate significant differences by Tukey’s test at the 0.05 level.

### Correlation between growth attributes and other parameters

GCI had no relationship with the biomass of any plant part or with N and P nutrient levels (Table 3). LA had a positive relationship with shoot and whole-plant biomass and most of the N and P nutrient levels. Height had a positive relationship with shoot P concentration and content and whole-plant P content. Again, RCD had no relationship with any of the biomass or nutrient parameters.

**Table 3:** Relationship between morphological traits and biomass, N and P concentrations, and N and P contents in *Podocarpus macrophyllus* seedlings.

Parameters for forecasting	Coefficients	GCI	LA	Height	RCD
**Shoot Biomass**	*r*	-0.550	0.867	0.665	0.512
	*P*	0.158	0.005	0.072	0.194
**Whole-plant Biomass**	*r*	-0.557	0.861	0.700	0.514
	*P*	0.152	0.006	0.053	0.192
**Shoot N concentration**	*r*	-0.613	0.659	0.605	0.582
	*P*	0.107	0.076	0.112	0.130
**Whole-plant N concentration**	*r*	-0.580	0.628	0.579	0.539
	*P*	0.132	0.096	0.133	0.168
**Shoot P concentration**	*r*	-0.705	0.856	0.720	0.620
	*P*	0.051	0.007	0.044	0.101
**Whole-plant P concentration**	*r*	-0.657	0.715	0.6638	0.590
	*P*	0.077	0.046	0.073	0.123
**Shoot N content**	*r*	-0.660	0.821	0.681	0.623
	*P*	0.075	0.013	0.063	0.100
**Whole-plant N content**	*r*	-0.657	0.806	0.706	0.615
	*P*	0.077	0.016	0.050	0.105
**Shoot P content**	*r*	-0.667	0.919	0.724	0.609
	*P*	0.071	0.001	0.042	0.110
**Whole-plant P content**	*r*	-0.698	0.875	0.755	0.638
	*P*	0.054	0.005	0.030	0.089

Values in light green background cells are significant regressions.

GCI, leaf green color index; LA, leaf area; RCD, root-collar diameter.

## Discussion

According to the classical dose-response model, nutrient status can be divided into three types, deficiency, luxury consumption, and toxicity, with the increase in the nutrient supply [27]. These nutritional statuses can be characterized by the synthesized responses of biomass, nutrient content, and nutrient concentration to a range of fertilization rates, which were all identified by vector analysis in our study.

### The characteristics of nutrient deficiency status

In our study, the nutrient deficiency status ranged between 0 and 60 mg N seedling^-1^ for Buddhist pine seedlings because shoot biomass increased in this treatment compared to that in the control; this status was also confirmed by the vector analysis. The critical rate of 60 mg N seedling^-1^ as the upper limit of the deficiency status in the Buddhist pine seedlings in our study was higher than that found for seedlings of *Picea mariana* (30 mg N seedling^-1^) [27] and *Quercus rubra* (25 mg N seedling^-1^) [28]. Our critical value was also close to that found for *Betula alnoides* (50 mg N seedling^-1^) [14], and lower than that for *Q*. *ilex* (150 mg N seedling^-1^) [29]. The differences among studies were generated by the varying efficiency of biomass accumulation at a given nutrient uptake speed depending on the tree species.

We also found increased height and root length and surface with the increase in the EF rate from 0 to 60 mg N seedling^-1^ without any changes in RCD. In the deficiency status, height was also found to be increased with fertilization in *P*. *mariana* [27] and *B*. *alnoides* [14]. In contrast, in deficient *Q*. *rubra* seedlings, height was unaffected, but RCD increased with fertilization [28]. Therefore, height and RCD appeared to be less reliable for forecasting the deficiency status than biomass. In accordance with our study, fine root length also showed a slight increasing trend in *Catalpa bungei* clones with increasing EF rates [30]. Uscola *et al*. [29] also found an increasing trend in total root volume in response to EF in the deficiency status. Therefore, fine root development can be taken as one of the typical responses of tree seedlings to the increase in the fertilizer dose in the deficient status. Our results were reasonable because it was found that when coniferous seedlings were facing the N-deficient condition, the addition of N would enhance uptake by promoting fine root growth [31].

### The characteristics of luxury consumption status

The luxury consumption state for a growing tree seedling was generated by the steady-state uptake of nutrients without remarkable changes in biomass [7]. This was caused by the natural symptoms of tree seedlings stopping growth in height and RCD and biomass accumulation after the nutrient deficiency is satisfied while their roots are still working to absorb nutrients [32]. The range of EF rates that induced this nutritional status was from 60 to 120 mg N seedling^-1^ in our study. The ranges of fertilization rates that led to the luxury consumption status were 30‒64 mg N seedling^-1^ in *P*. *mariana* [27], 25‒100 mg N seedling^-1^ in *Q*. *rubra* [28], 150‒200 mg N seedling^-1^ in *Q*. *ilex* [29], and 50‒400 mg N seedling^-1^ in *B*. *alnoides* [14]. These fertilizer-dose ranges were the result of preconditioning both doses to define deficient and optimum growth states. Within the luxury consumption status, the root morphology parameters and scanned leaf indices all remained constant as well. These results suggest that the uptake of nutrients during luxury consumption did not require a fine root growth response. However, our R/S values showed an increasing trend with the increase in the EF rate in this status. In contrast, former studies reported R/S to be either unchanged [27,28] or to decline [14,29] in this status. Buddhist pine is an ancient, slow-growing tree species, and its shoot growth does not show apparent changes in one month unless it is placed under an extended photoperiod [25]. Therefore, the increased R/S in the luxury consumption status could facilitate high resource utilization efficiency in this species.

### Excessive nutrient supply symptoms

In our study, when EF was conducted on Buddhist pine seedlings at rates higher than 120 mg N seedling^-1^, the symptoms of excessive nutrient supply occurred. Our results concurred with findings by Wei *et al*. [25], who reported that Buddhist pine seedlings showed toxicity symptoms in the EF treatment at a rate of 150 mg N seedling^-1^ but not in that of 100 mg N seedling^-1^. On the basis of luxury status, the toxicity dose at the lowest level was different among species in the range from 25 mg N to 400 mg N per seedling [14,27–29]. With the biomass decline, all growth and morphological parameters decreased as well. An experiment on mined land revealed that the negative effects of nursery-induced excessive nutrient supply on seedling growth and biomass accumulation persisted for as long as a year [33].

### Forecasting nutrient status using scanned leaf indices

Digital scanning and analysis technology make it possible to rapidly forecast seedling status at a high efficiency using leaf images. In our study, GCI had no relationship with either N or P concentration in the shoot or root. In contrast to our results, Rabara *et al*. [18] found some interplay between GCI and chlorophyll content in artichoke seedlings. Zhu *et al*. [19] found a negative relationship between GCI and foliar N concentration in pepper plants. These contradictions probably existed because we determined the nutrient concentrations in the shoot part (leaves + stem) instead of in the leaves, but GCI is a parameter measured in leaves. Using both organs resulted in the failure to relate nutritional parameters to GCI. Therefore, GCI was not a proper parameter for forecasting nutrient concentration in shoot organs at different nutrient statuses. GCI may be available to predict leaf nutrition which, however, we did not detect leaves. In this regard, we do not recommend GCI for forecasting nutritional status in seedling parts, including woody organs.

In our study, LA was found to have a positive relationship with root morphologies, which concurred with findings by Reich *et al*. [34] driven by the coupling effect at the tissue level. The positive relationship between LA and root morphologies was in accordance with McDowell *et al*. [35], where foliar and root morphologies had an association with seizing light and water resources, respectively. We also found positive relationships between LA and shoot biomass and between LA and shoot P concentration. Similar results were reported in a study on 79 perennial plants; the underlying mechanism was that larger LA values resulted in higher respiration and transpiration, which promoted P uptake through upward sap flow and thereafter photosynthetic production [36]. We did not find a relationship between LA and N concentration. This suggests that the expansion of leaf size did not depend on N allocation to leaves. Further studies should be conducted to clarify this relationship for broadleaf species.

### Forecasting nutrient status from growth attributes

Height and RCD have been employed as growth attributes for forecasting seedling quality and transplant performance in at least 30 trials [5,6]. Our findings showed that seedling height was positively related to shoot P concentration. Therefore, height had a further positive relationship with shoot P content because nutrient content is the product of concentration and biomass. Because of the shoot P uptake relationship with height growth, there was another positive relationship between height and whole-plant P content. These results agreed with the results found by Wang *et al*. [37] but contradicted those of Brown [38]. With nutrient delivery through EF, P can be highly utilized and preserved by seedlings in accordance with their height growth.

Root morphology was one of the parameters for evaluating seedling quality that was tested at the end of nursery culture [38]. The development of the root system has been found to forecast transplant performance in nursery-cultured seedlings [39]. In this study, root morphologies were found to be positively correlated with N and P uptake to the shoot part, suggesting the promotion of efficient nutrient uptake through root morphology development. The positive relationship between root morphology and nutrient uptake in mature trees has also been documented in previous studies [40,41]. Under conditions where environmental factors are well controlled, assessing root morphology can be used as an approach to more quickly and precisely indicate nutrient concentration levels than that of chemical analysis.

## Conclusions

In this study, Buddhist pine seedlings were raised at a range of EF rates from 0 to 140 mg N seedling^-1^ in multishelves for which most environmental factors were highly controlled. Seedlings were determined to have different nutrient statuses with different growth, morphology, biomass, and nutrient response parameters. According to the changes associated with these parameters, the nutritional responses of Buddhist pine seedlings were graded into statuses of deficiency, luxury consumption, and toxicity in response to dose-ranges of 0–60 mg N seedling^-1^, 60–120 N seedling^-1^ and over 120 N seedling^-1^, respectively. Morphological traits, GCI and LA, obtained by digital analysis also had significant responses to the range of EF doses, but only LA had a positive relationship with most nutritional parameters. Therefore, a dose range between 60 and 120 mg N seedling^-1^ was recommended for the culture of Buddhist pine seedlings, and leaf area assessment through digital scanning can be used as an easy and fast approach for indicating inherent nutrient status.

## Supporting information

S1 Data (XLSX)
